# Supplementary material for: A Stress-Adaptive Lipid Kinase Axis Defines Metabolic Vulnerabilities in Neuroendocrine Prostate Cancer
Source: bioRxiv. 2026 Jun 18:2026.06.15.732391. Preprint. [Version 1] doi: 10.64898/2026.06.15.732391 (PMC13411104; doi:10.64898/2026.06.15.732391)

Figure S1. Related to Figure 1.

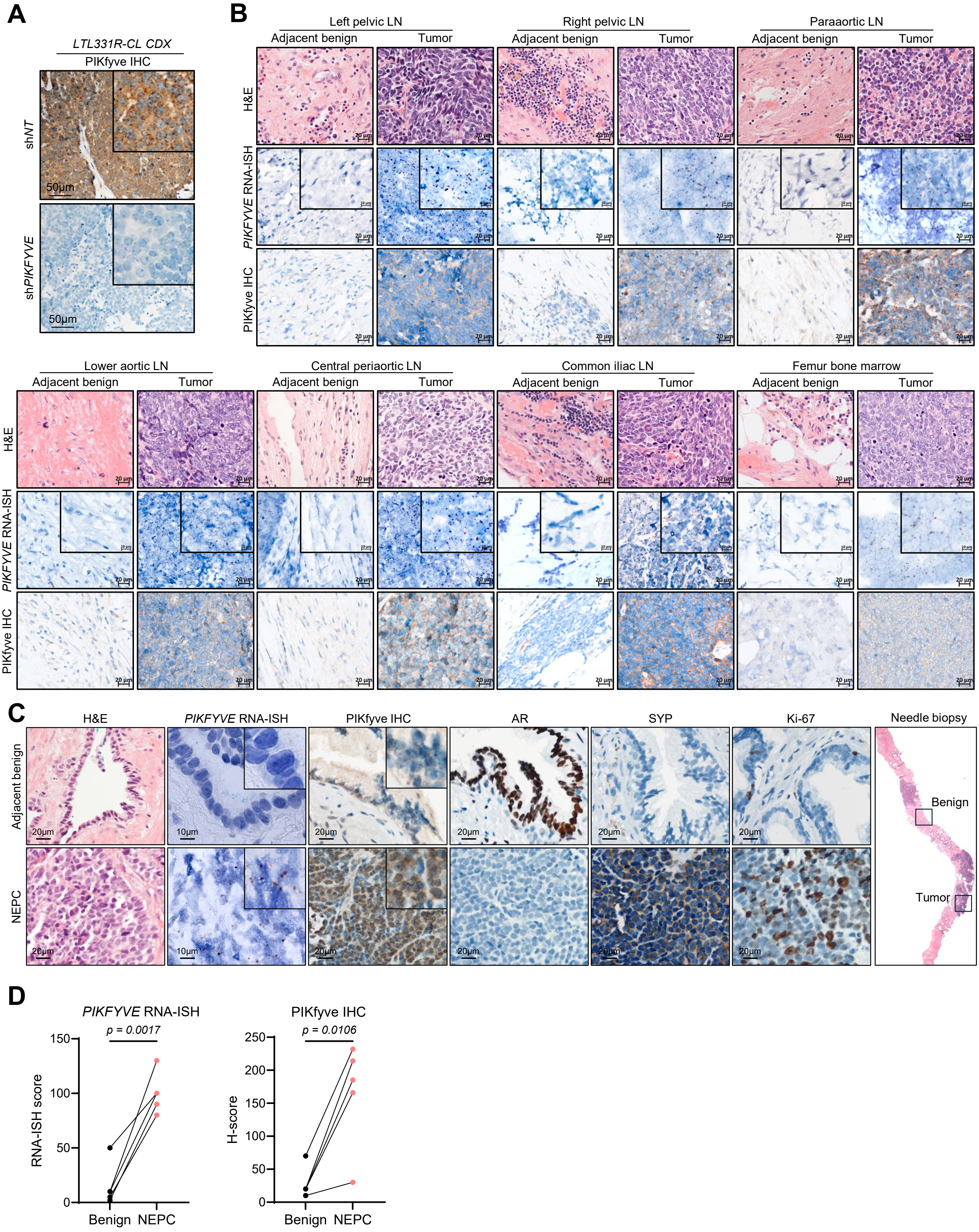

Figure S2. Related to Figure 1.

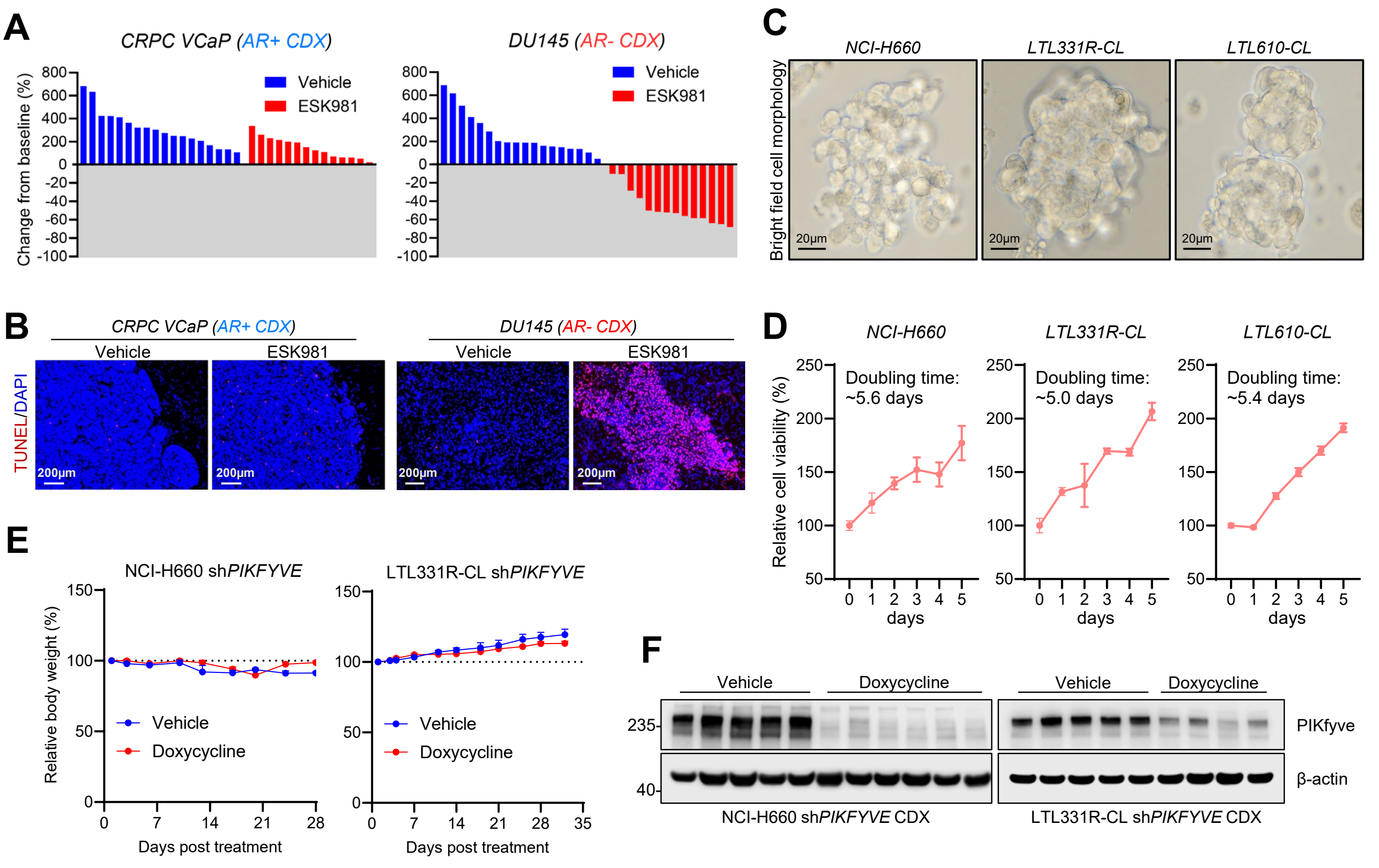

Figure S3. Related to Figure 2.

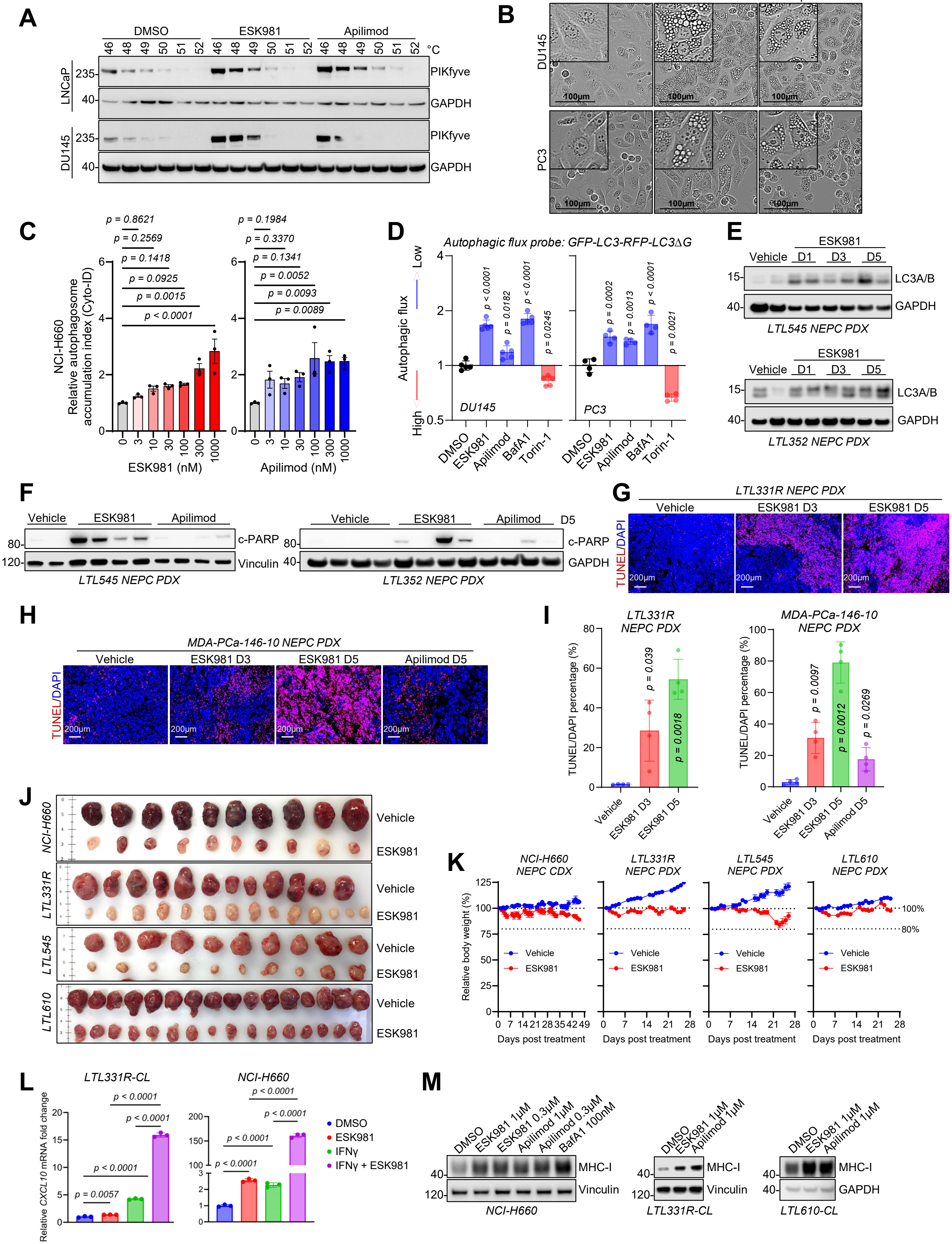

Figure S4. Related to Figure 3.

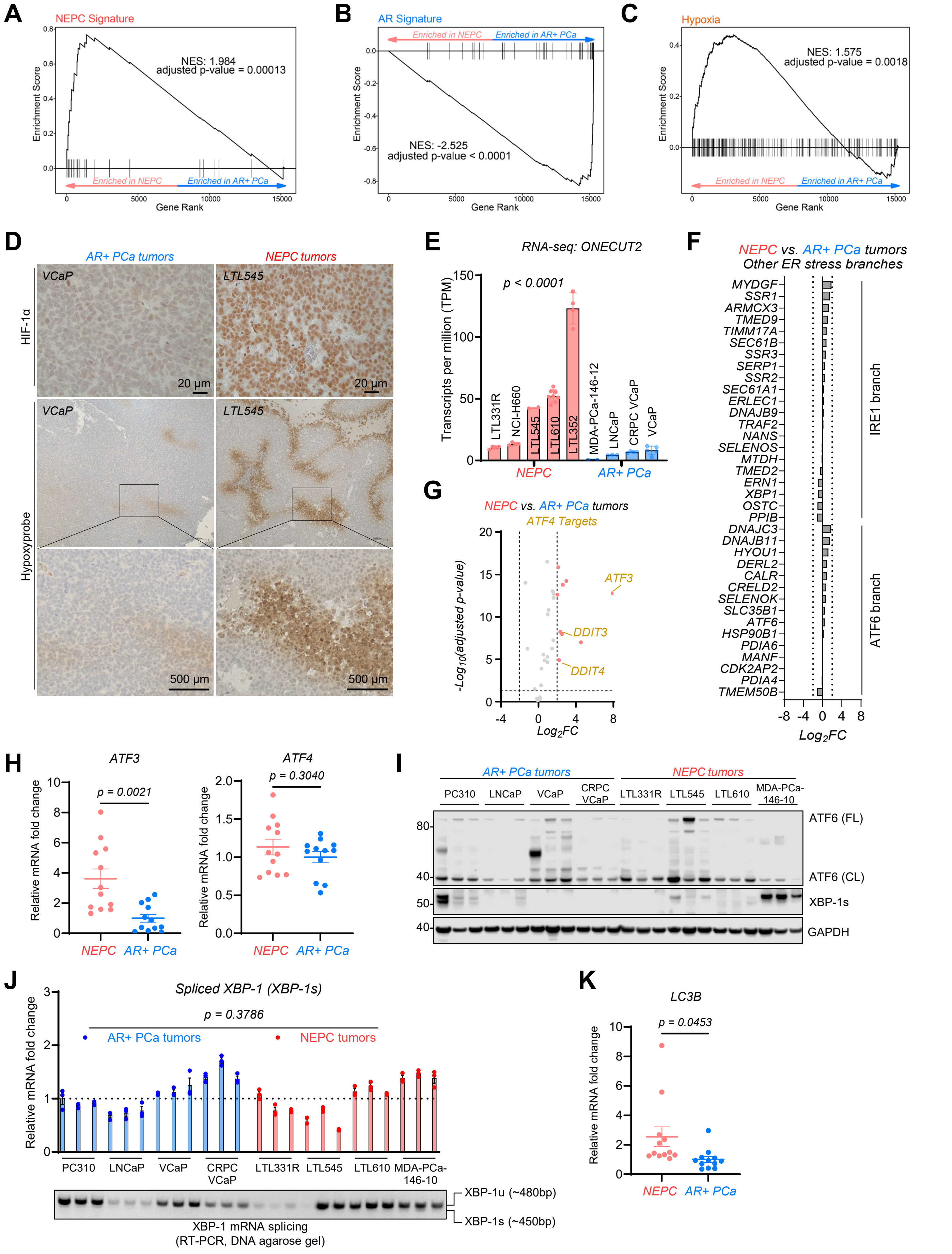

Figure S5. Related to Figure 4.

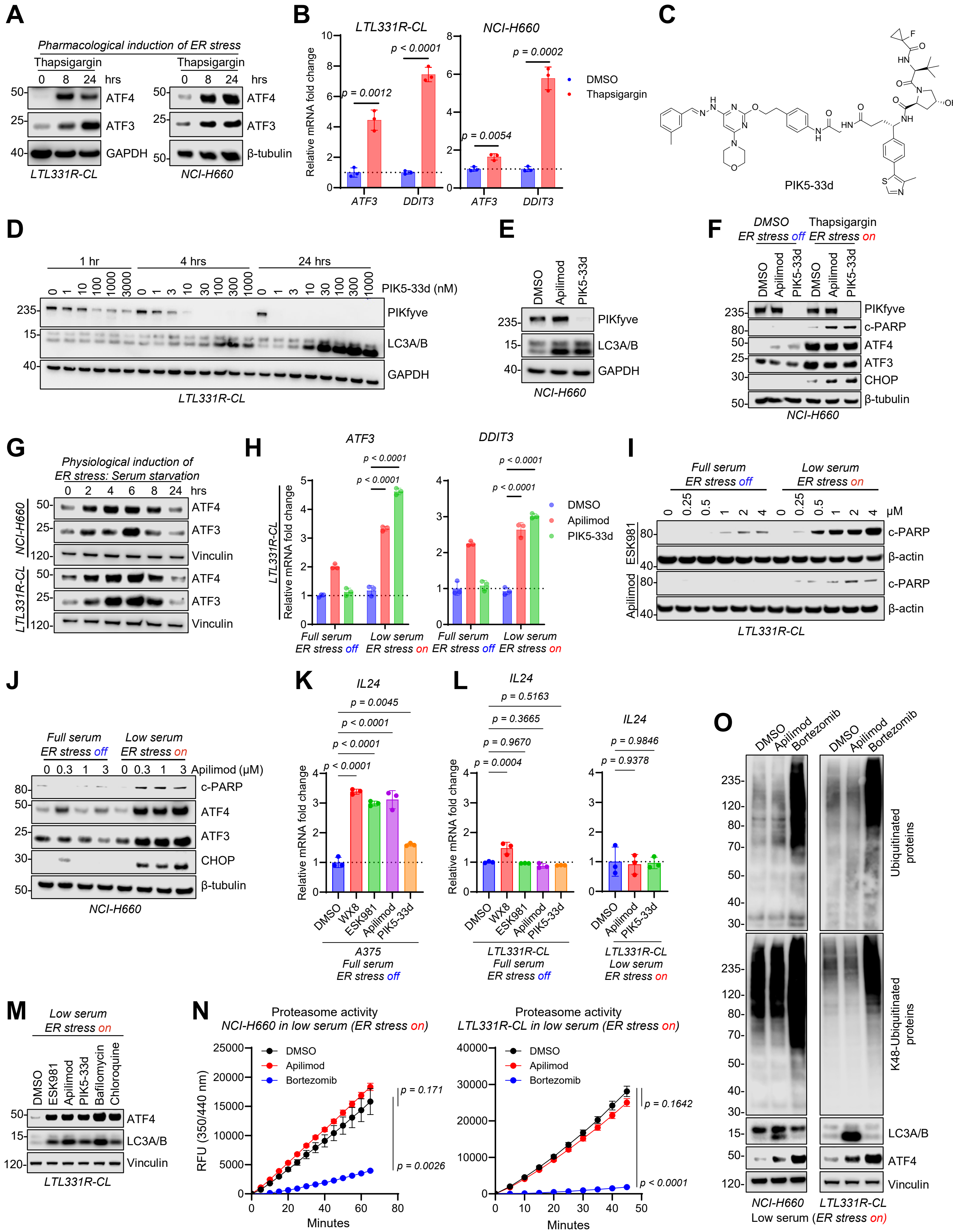

Figure S6. Related to Figure 4.

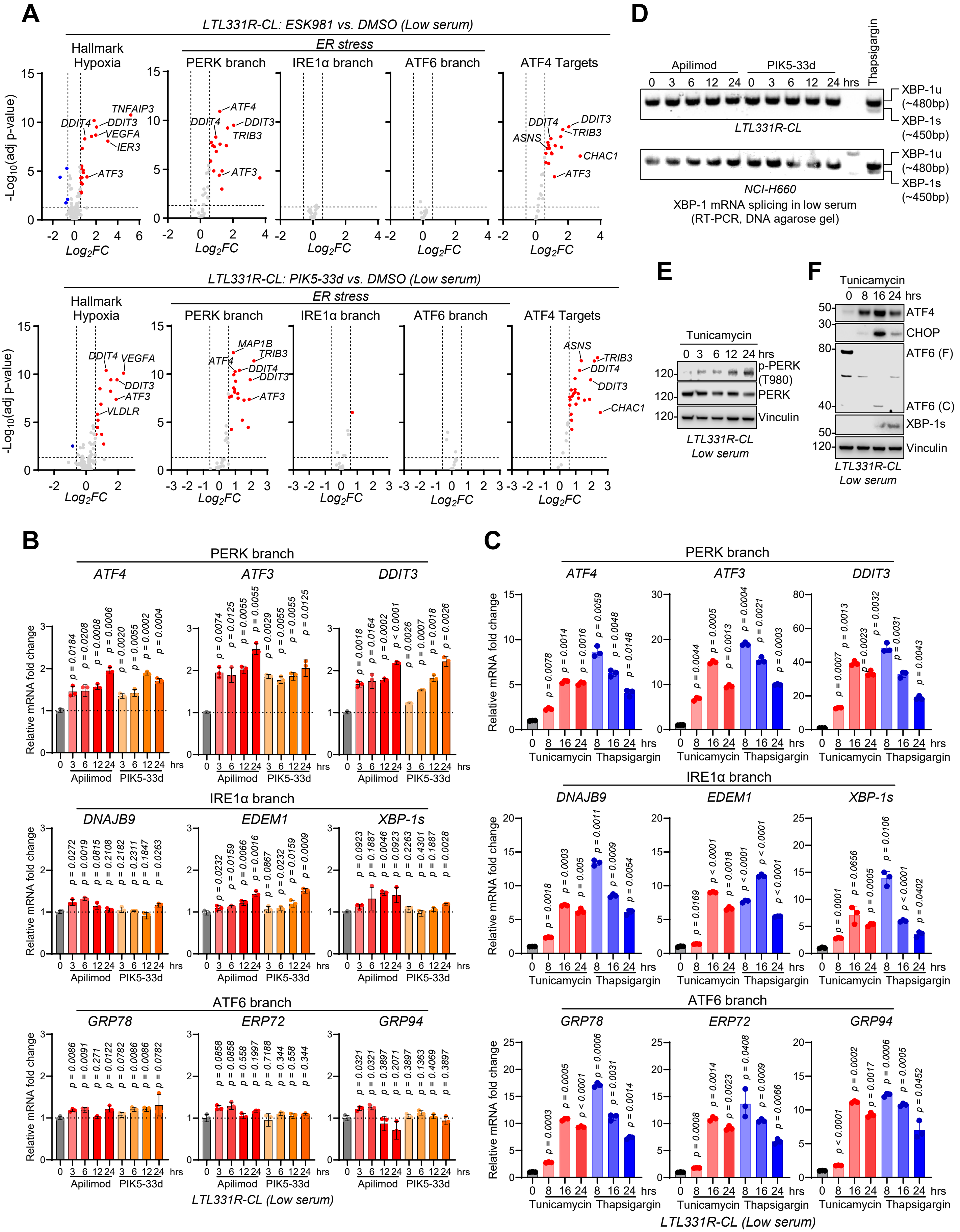

Figure S7. Related to Figure 4.

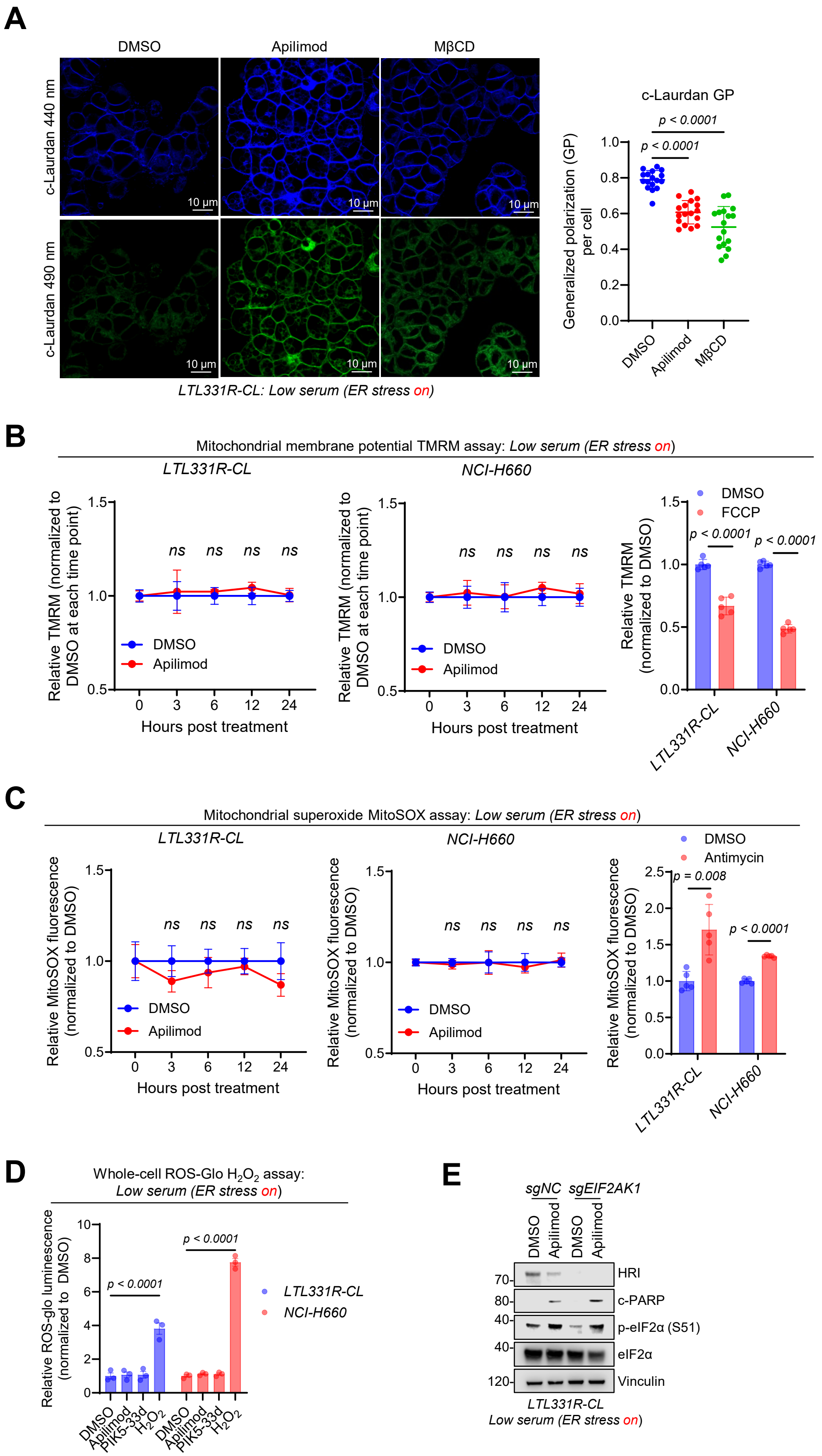

Figure S8. Related to Figure 4.

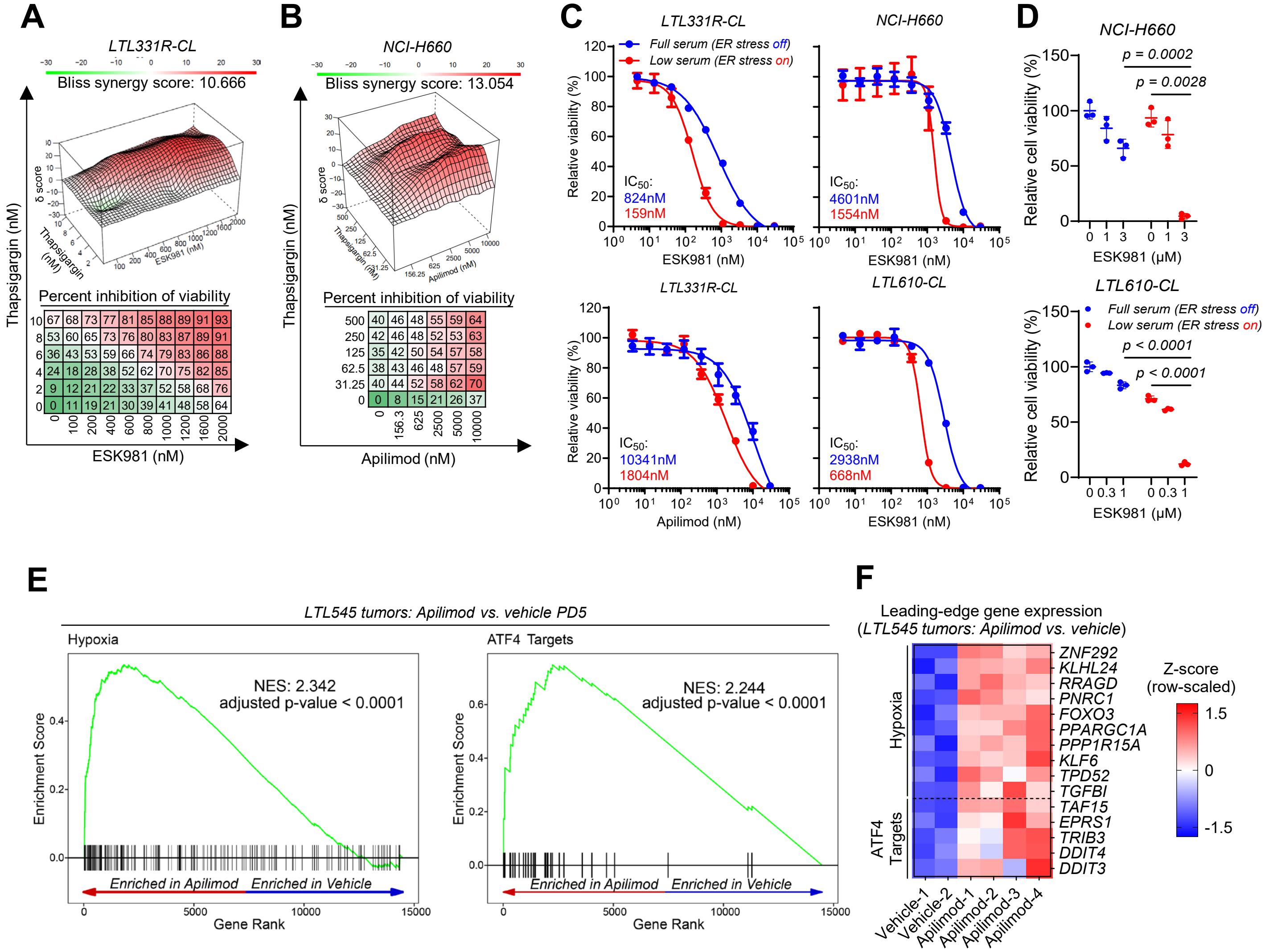

Figure S9. Related to Figure 5.

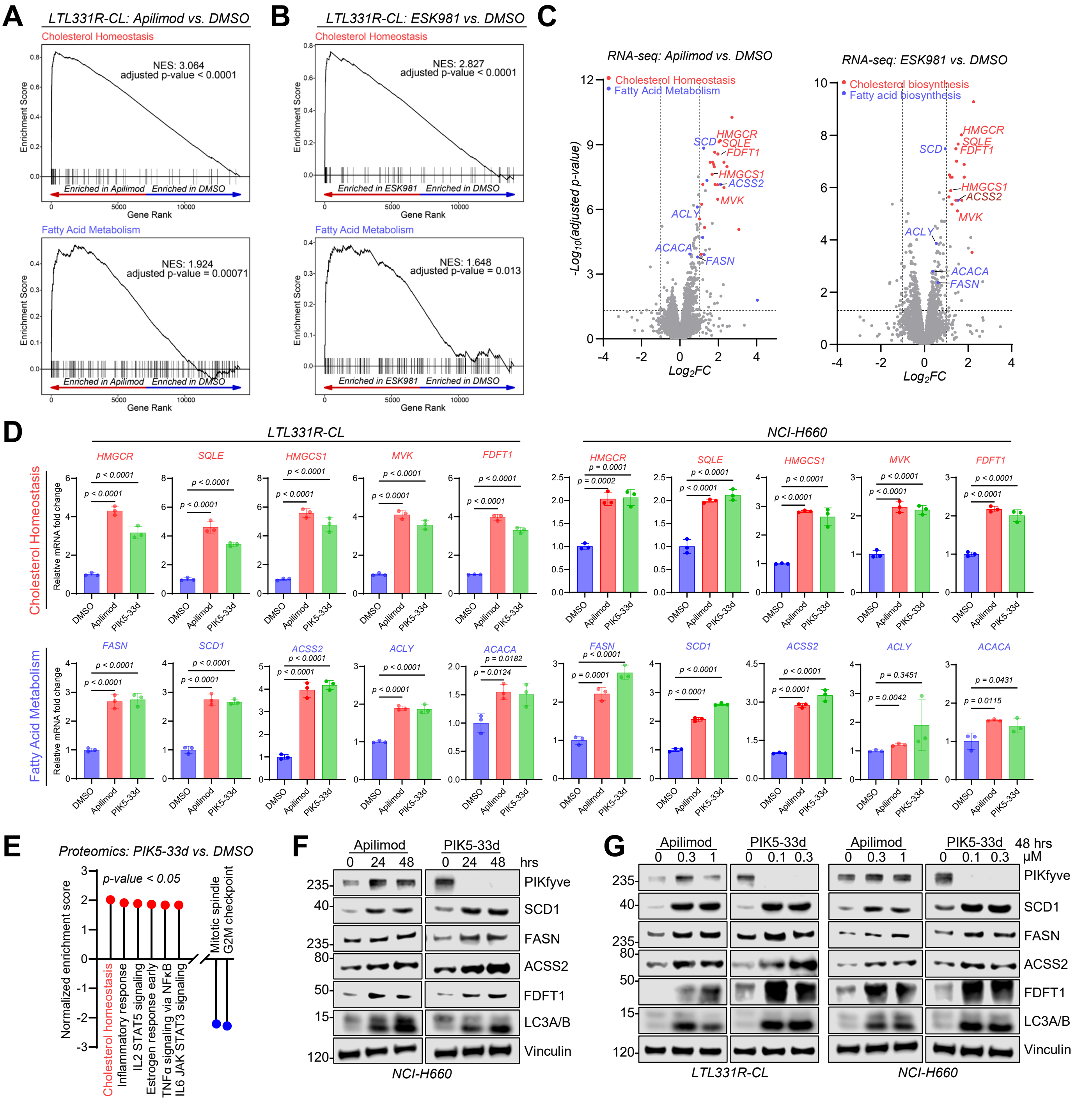

Figure S10. Related to Figure 5.

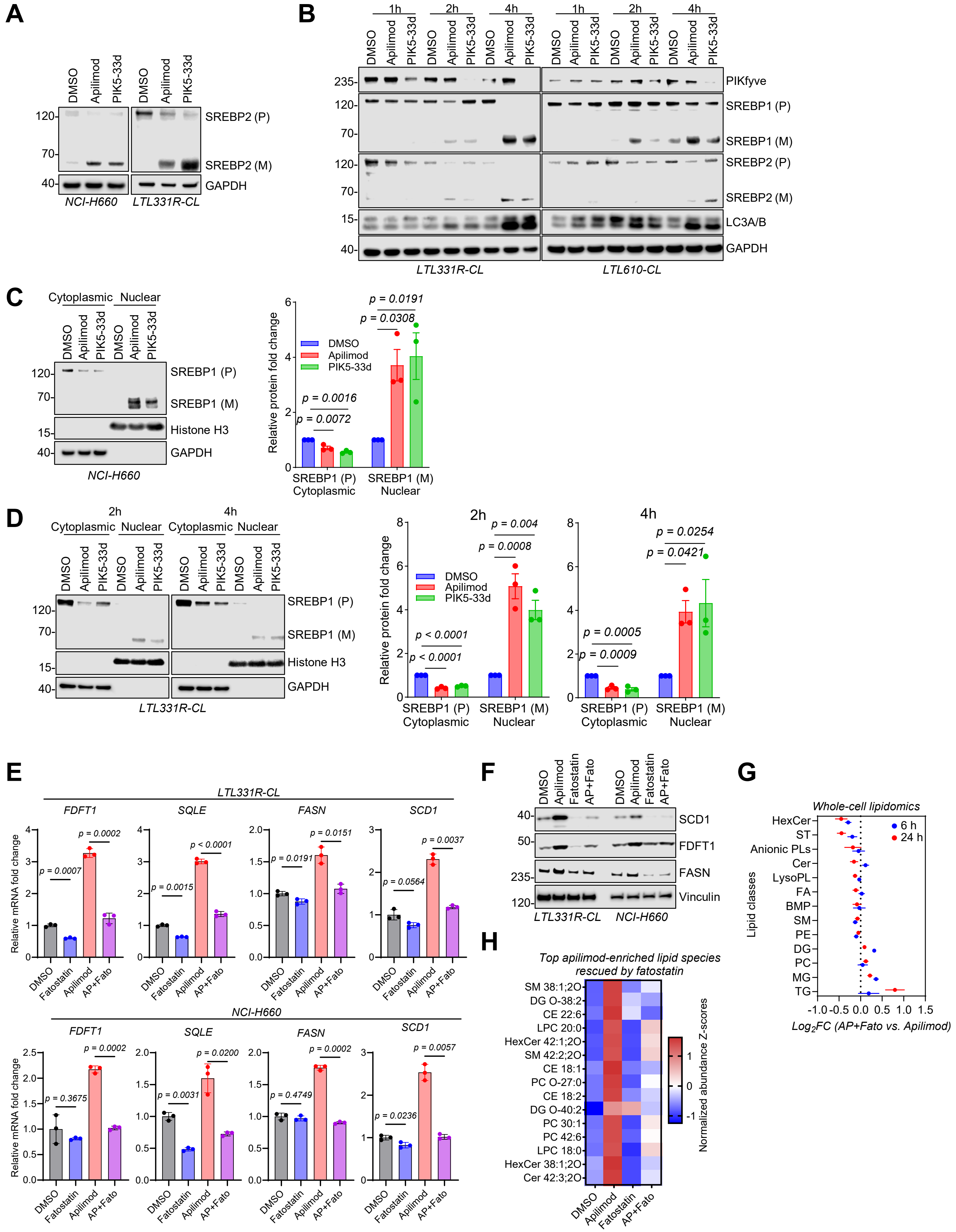

Figure S11. Related to Figure 5.

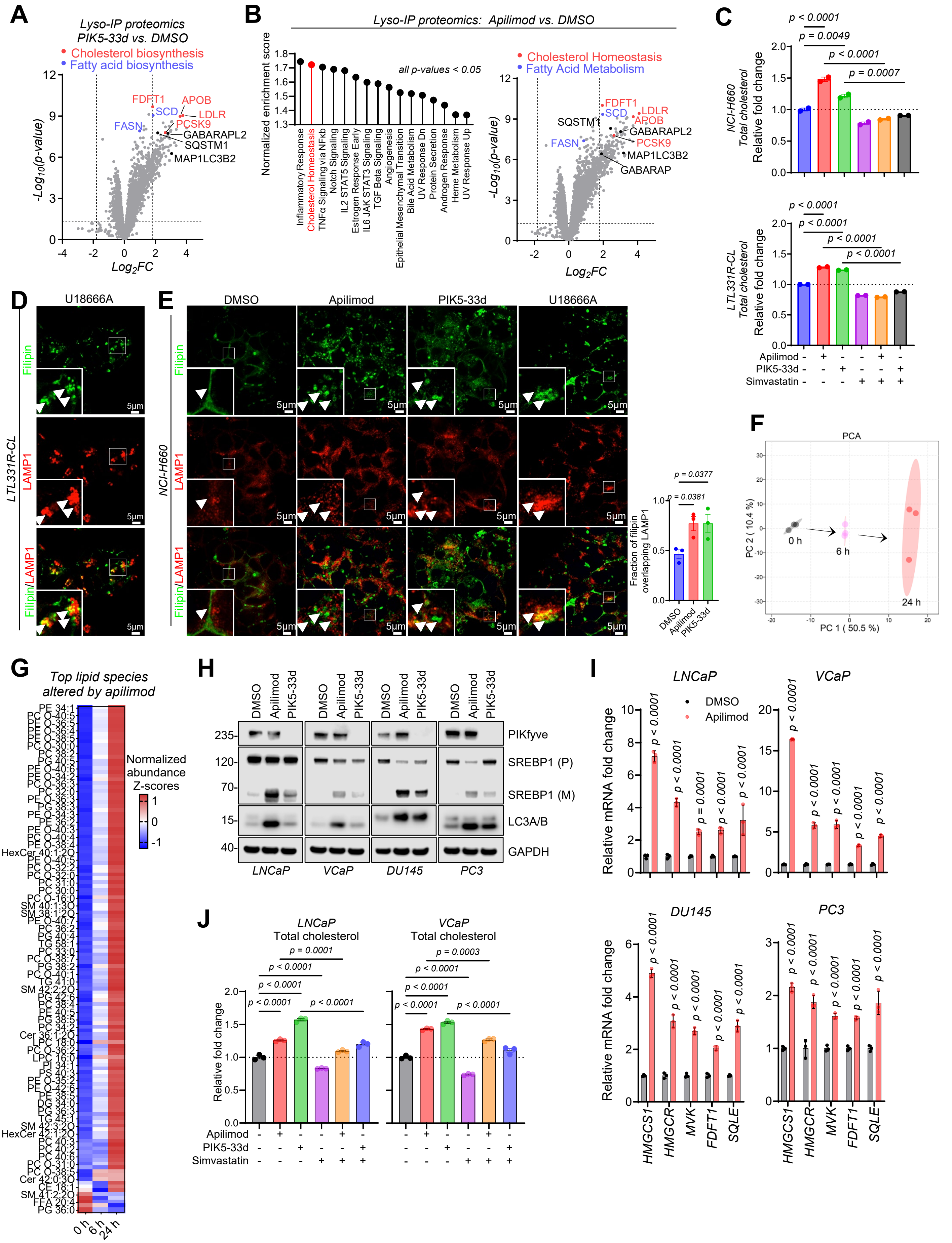

Figure S12. Related to Figure 6.

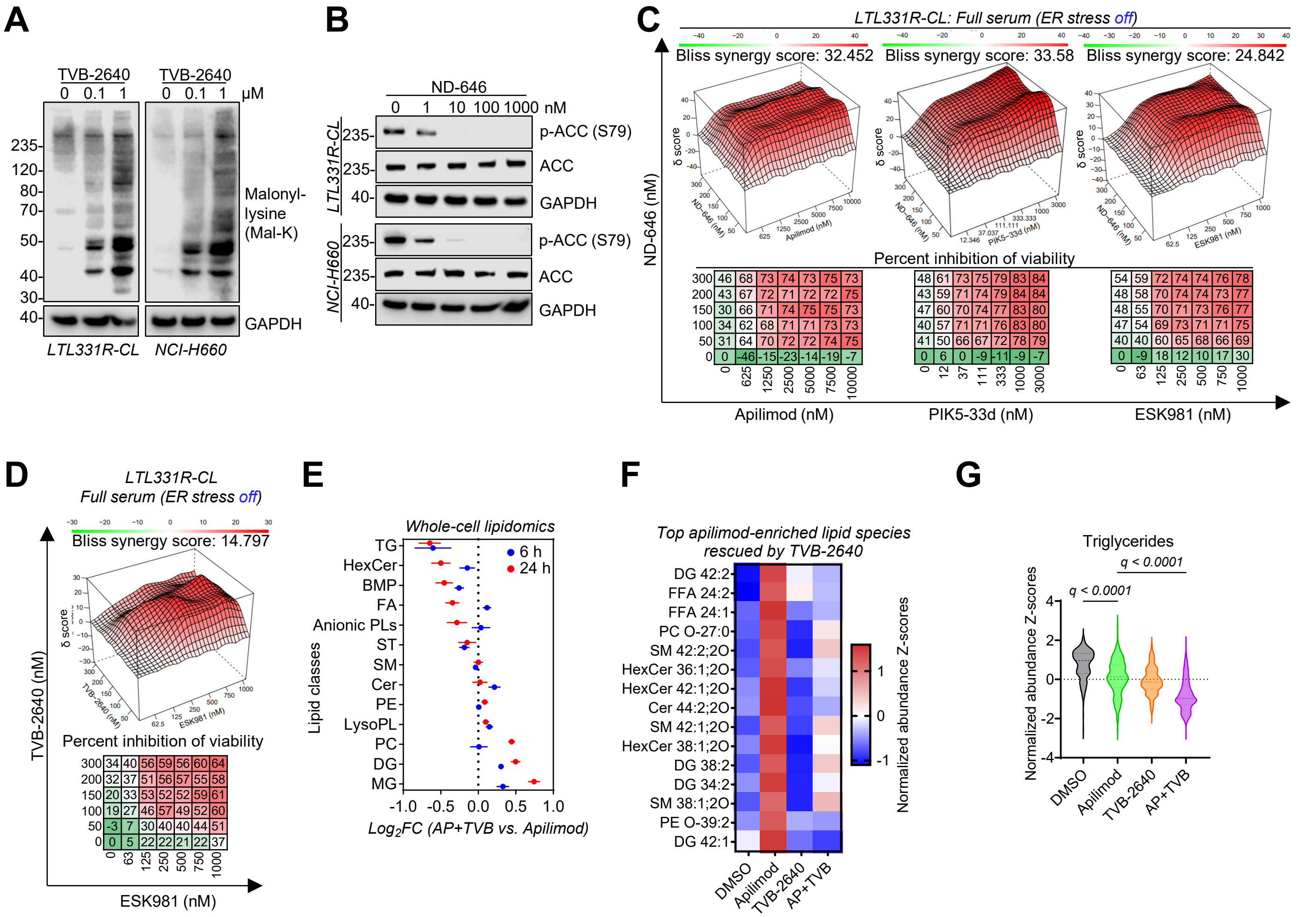

Figure S13. Related to Figure 6.

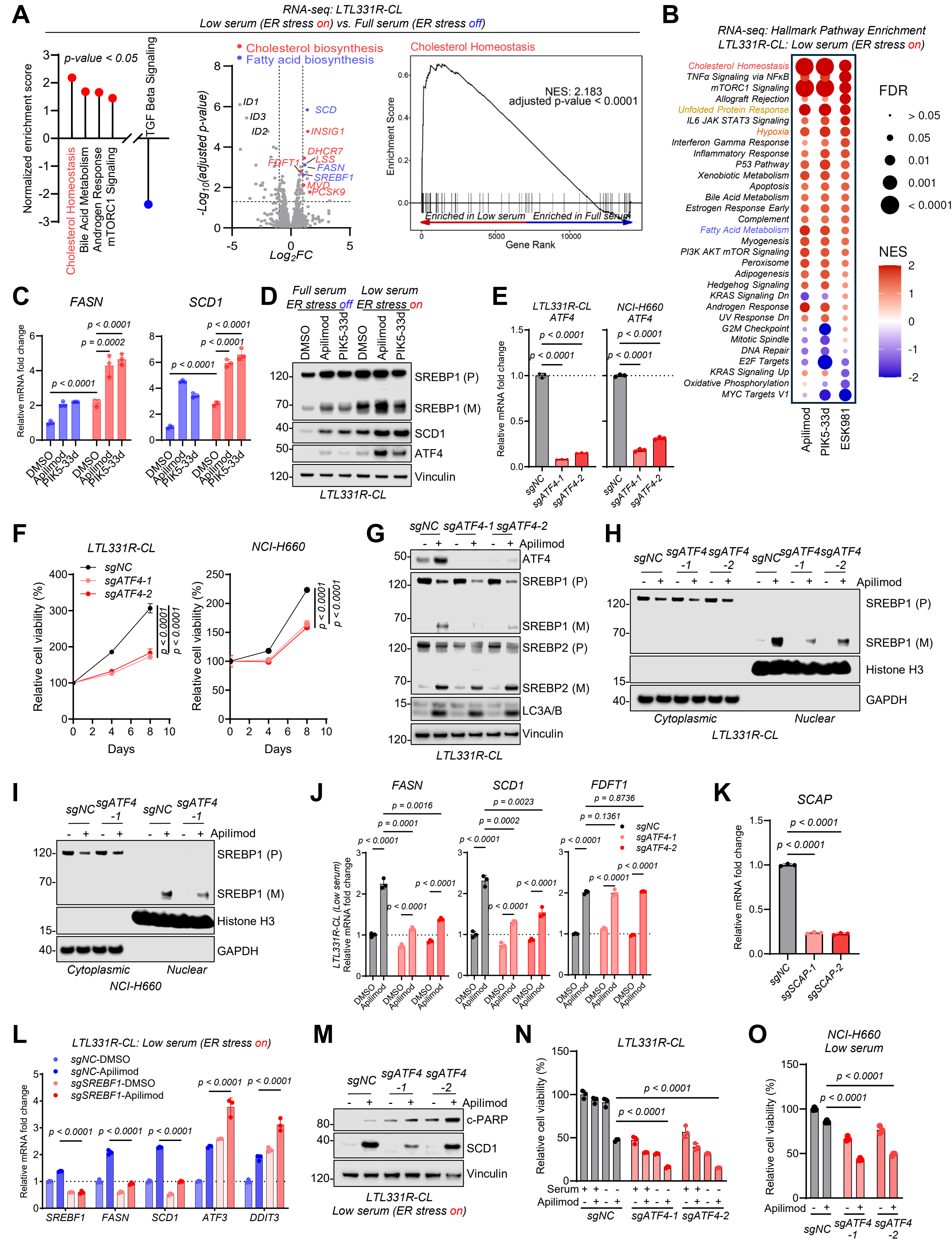

Figure S14. Related to Figure 6.

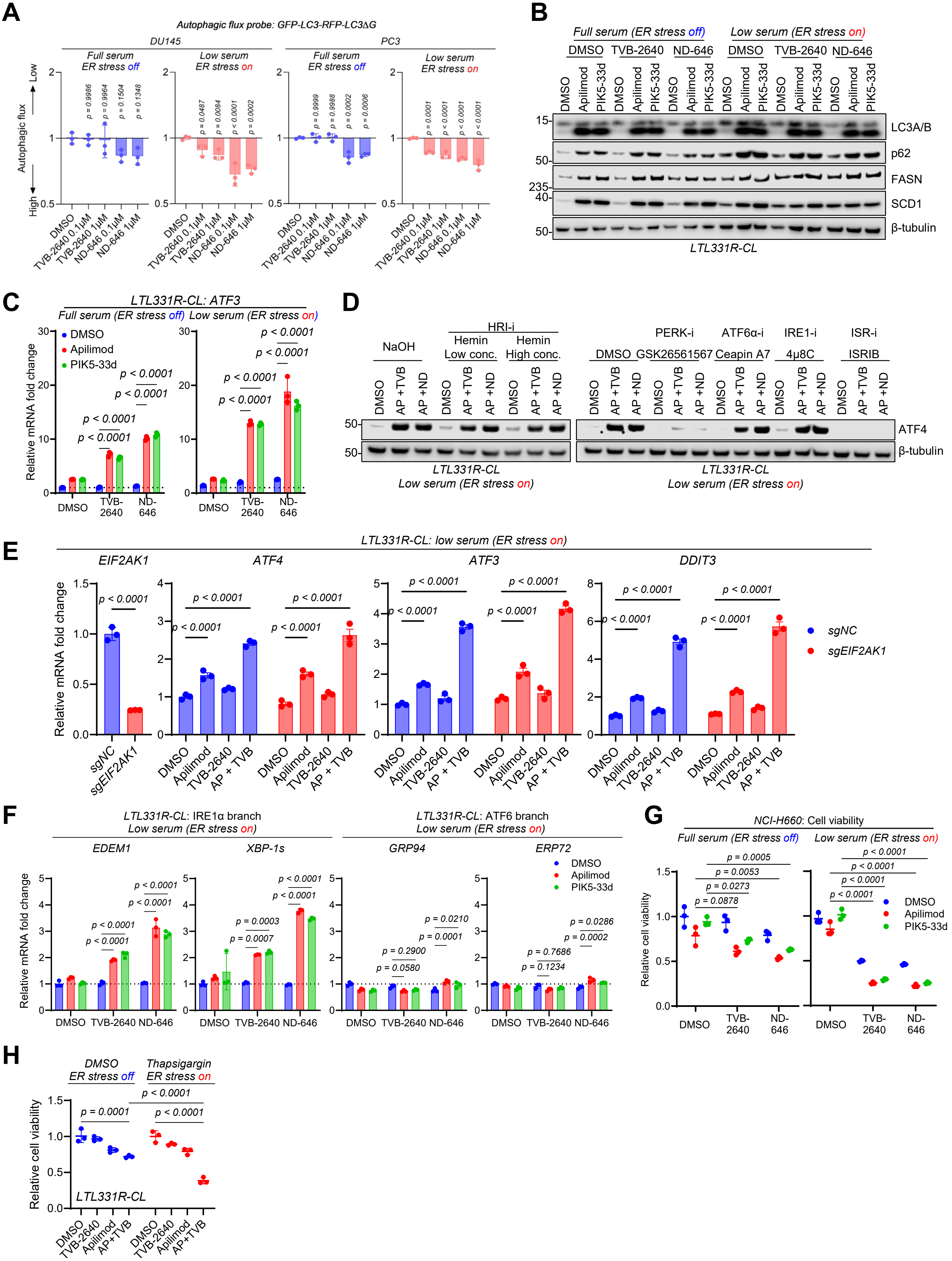

Figure S15. Related to Figure 6.

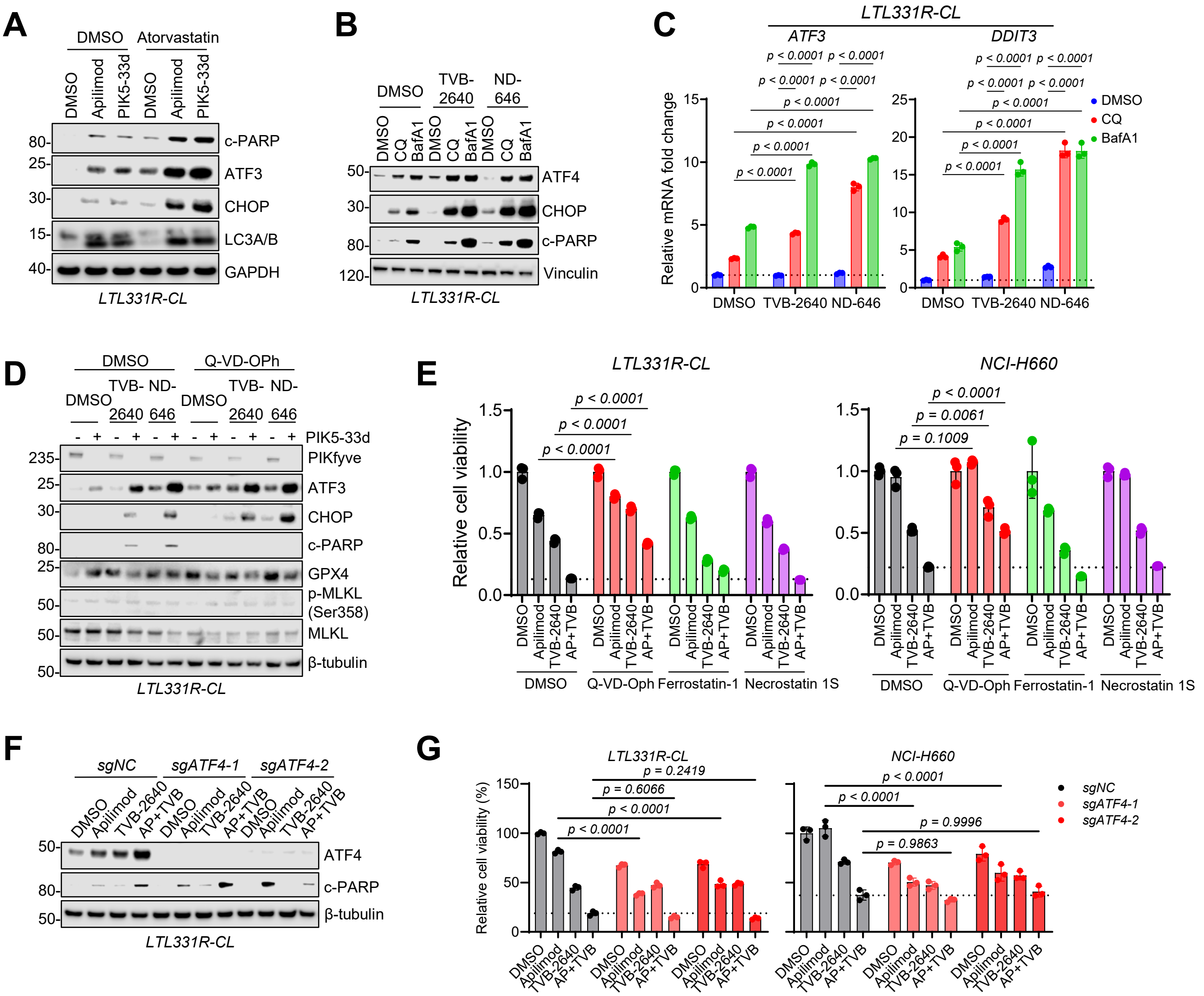

Figure S16. Related to Figure 6.

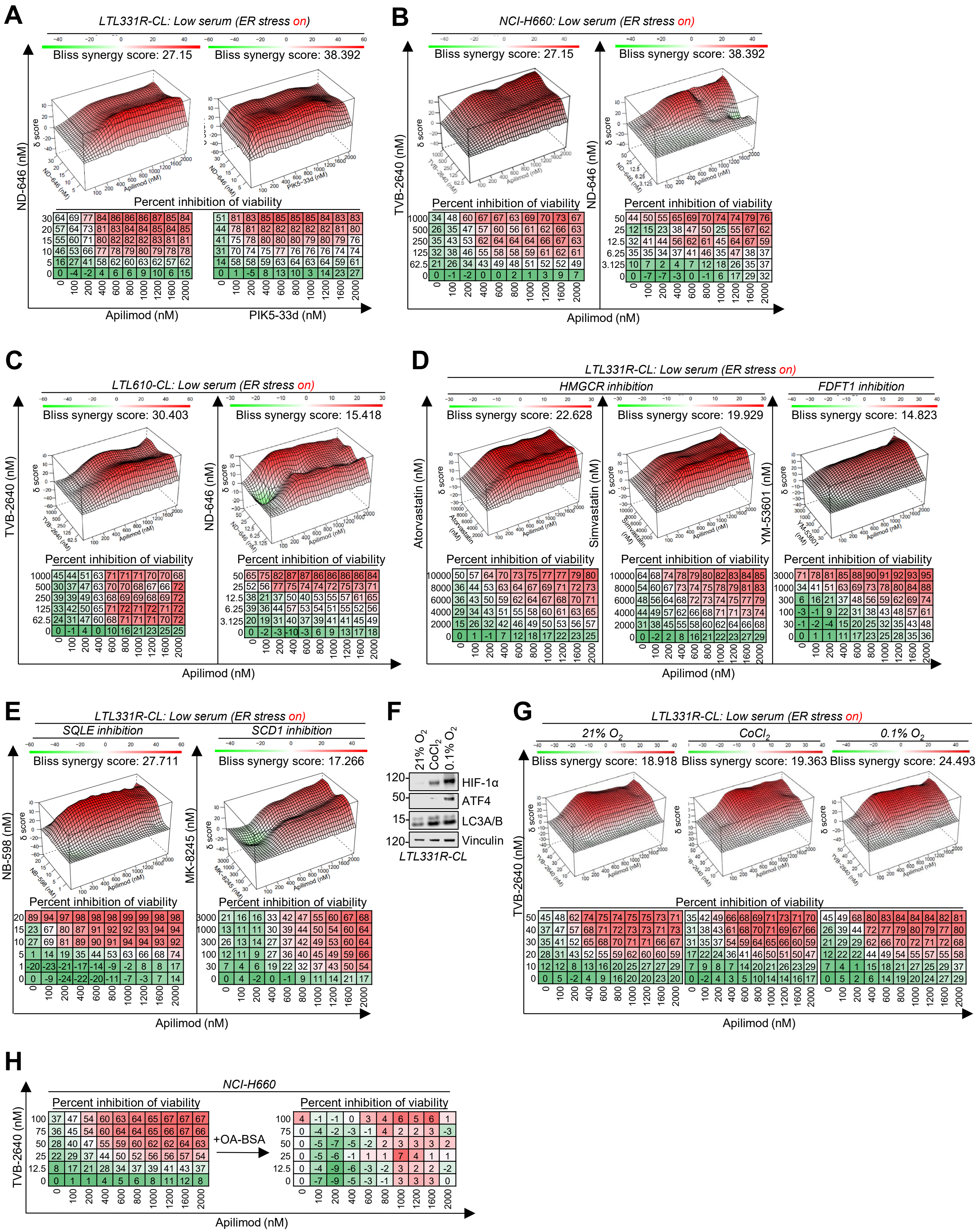

Figure S17. Related to Figure 7.

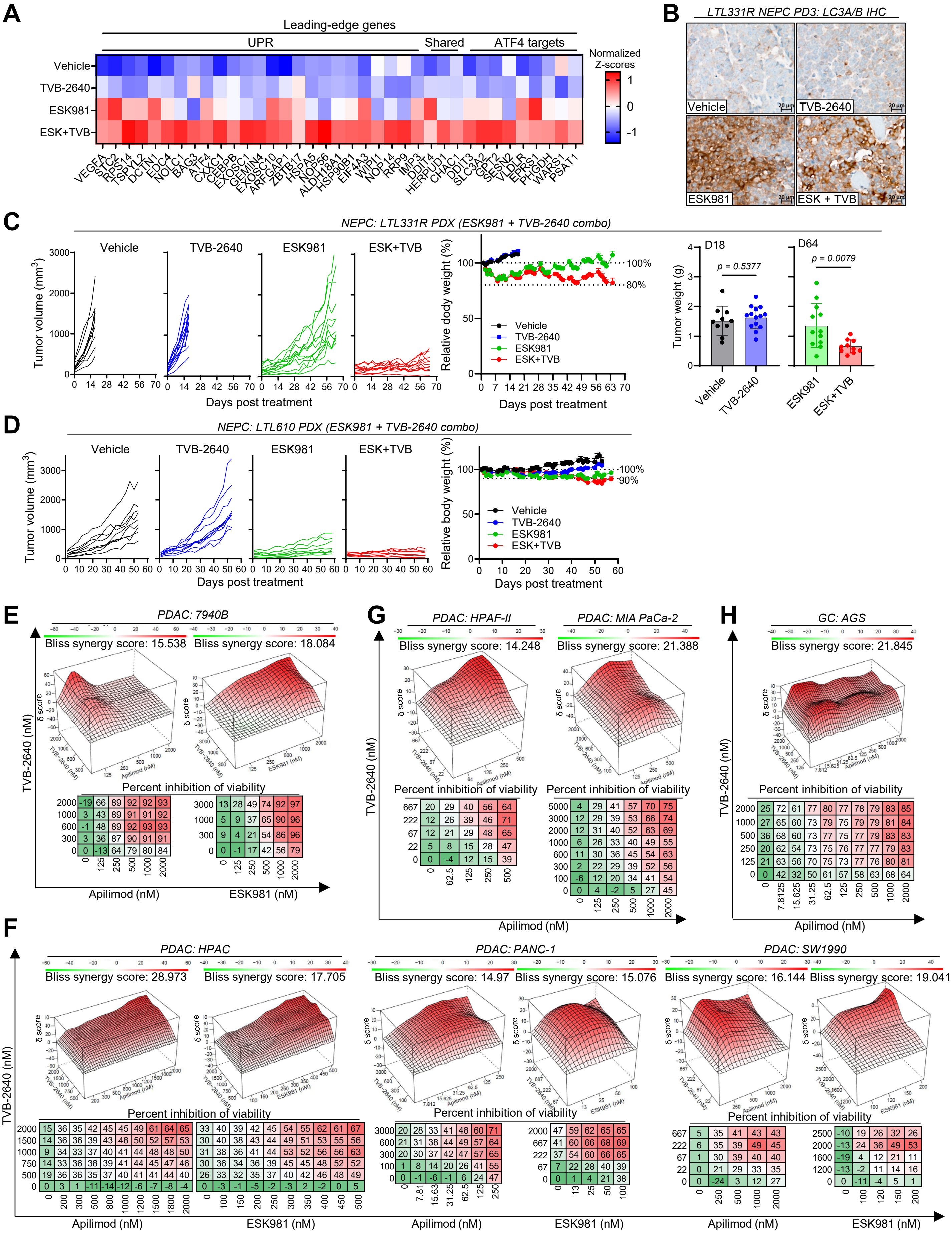

Figure S18. Related to Figure 7.

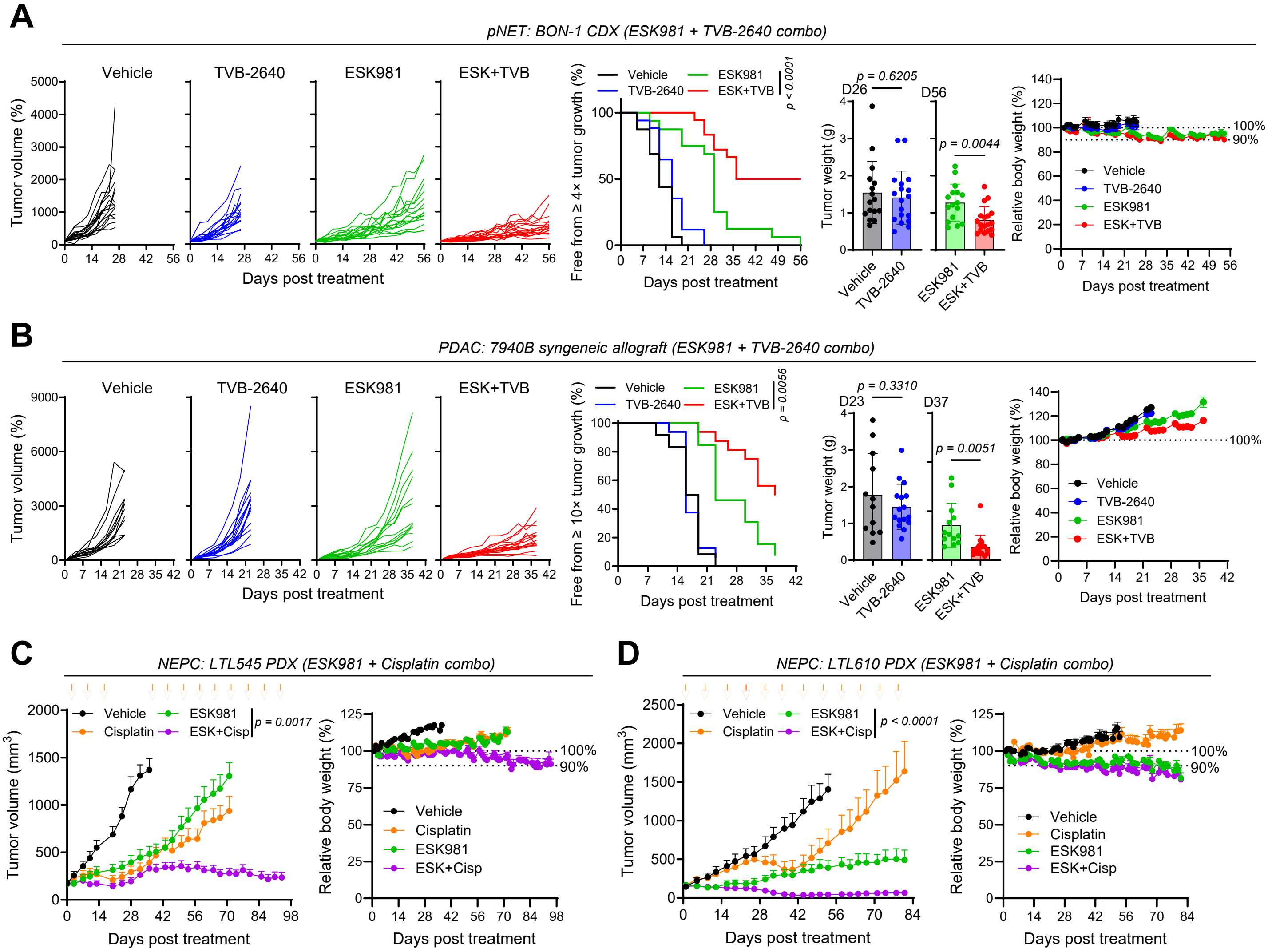

Figure S19. Related to Figure 7.

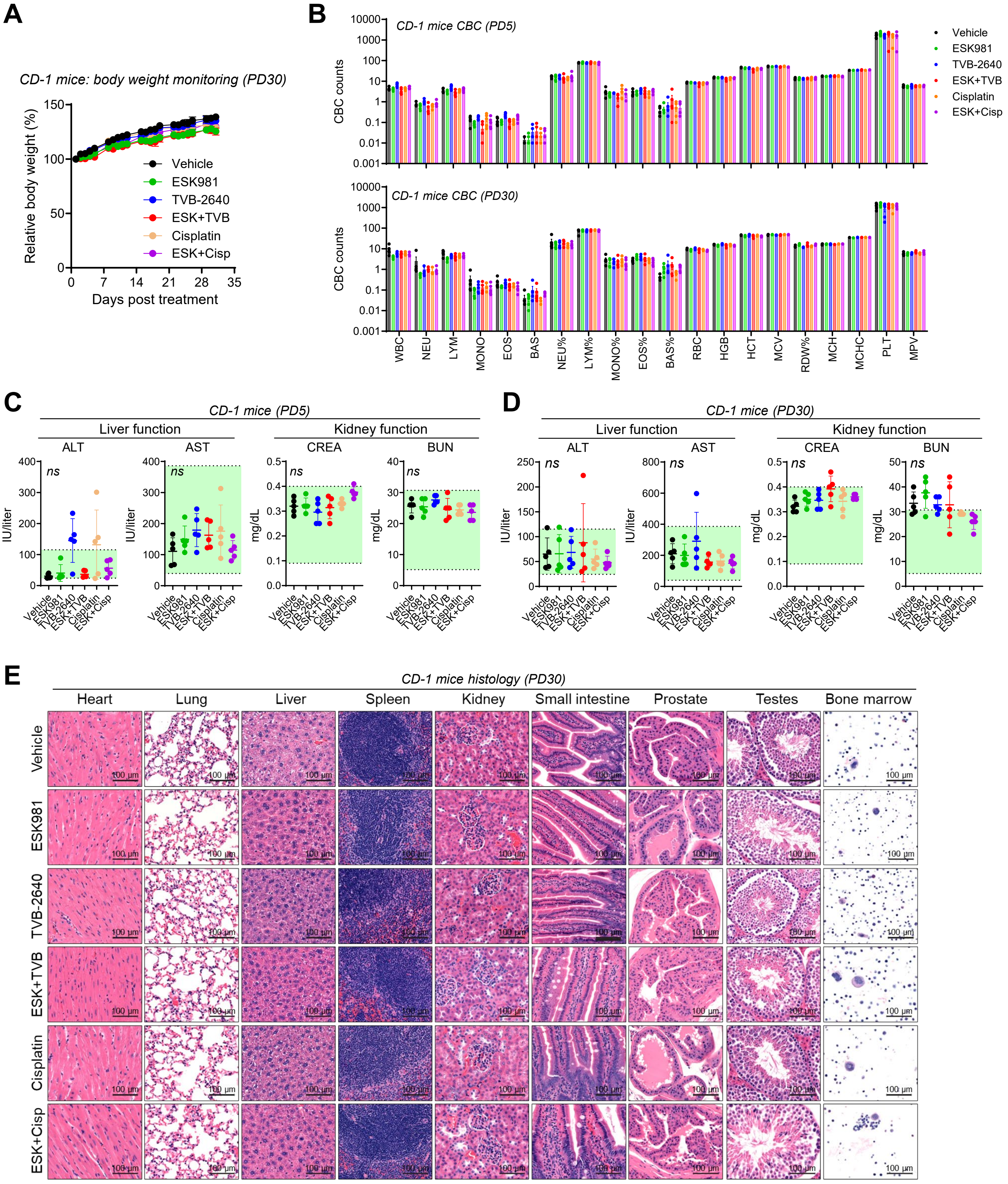

Supplement: Supplement 1 — Figure S1. PIKfyve is overexpressed in NEPC. Related to Figure 1. A. Representative images of PIKfyve IHC in shNT and shPIKFYVE tumors of LTL331R-CL NEPC CDX to validate the specificity of PIKfyve antibody. B. Representative histological and molecular images from remaining seven metastatic sites of WA76 case (Left pelvic LN, right pelvic LN, paraaortic LN, lower aortic LN, central periaortic, common iliac LN, and femur bone marrow), showing hematoxylin and eosin (H&E) staining, RNA in situ hybridization (RNA-ISH), and IHC for PIKfyve in matched tumor and adjacent benign tissues. C. Representative histology and molecular profiling of PIKfyve expression in five primary NEPC needle biopsy specimens. Shown are adjacent benign (top) and tumor (bottom) regions from the same biopsy core, stained by H&E, PIKFYVE RNA-ISH, and IHC for PIKfyve, AR, SYP, and Ki-67. Insets highlight higher-magnification views of RNA and protein localization. D. Quantification of PIKFYVE RNA-ISH score (left) and IHC H-score (right) across five needle biopsies between tumor regions and benign tissues. P values calculated using a paired two-tailed student’s t-test. Figure S2. PIKfyve serves as a therapeutic target in NEPC. Related to Figure 1. A. Waterfall plots showing individual tumor volume changes from baseline in vehicle or ESK981 treated tumors in DU145 (AR− CDX, vehicle n = 18, ESK981 n = 14) and CRPC VCaP (AR+ CDX, vehicle n = 18, ESK981 n = 14) tumors. Data adapted from Qiao et al., Nature Cancer, 202122. B. Representative images of TUNEL staining showing in situ cell death post five days of either vehicle or ESK981 treatment in DU145 (AR− CDX) and CRPC VCaP (AR+ CDX) tumors. C. Bright-field images showing morphology of NEPC ex vivo cell lines LTL331R-CL and LTL610-CL in comparison to NCI-H660. D. Doubling time of NCI-H660, LTL331R-CL, and LTL610-CL cells in vitro, determined by CellTiter-Glo. Data presented as mean ± SD. E. Body weight of mice bearing NEPC shPIKFYVE CDXs during the treatm [file media-1.pdf]
